# Supplementary material for: Nanowire-assisted electrochemical perforation of graphene oxide nanosheets for molecular separation
Source: Nat Commun. 2024 Jan 2;15:164. doi: 10.1038/s41467-023-44626-9 (PMC10762124; doi:10.1038/s41467-023-44626-9)
Supplement: Supplementary file 1 — Supplementary Information [file 41467_2023_44626_MOESM1_ESM.pdf]

Supplementary Information

Nanowire-assisted electrochemical perforation of graphene oxide nanosheets for molecular separation

Hai Liu[1], Xinxi Huang[1], Yang Wang[1], Baian Kuang[1], Wanbin Li[1]*

[1]School of Environment, Jinan University, Guangzhou, 511443, China.

*Corresponding author. Email: gandeylin@126.com

This file includes:

Supplementary Methods

Supplementary Figures 1 to 27

Supplementary Tables 1 to 4

Supplementary References 1 to 12

Supplementary Methods

Electrochemical properties of Co_3O_4-NW and Co_3O_4-NS anodes were characterized with a three-electrode system using 3.5 M Ag/AgCl as reference electrode with an electrochemical workstation (CHI760E, CH Instruments). Electrode potential distributions of the cathode and anode were recorded under applied voltages of 2–9 V with flow rate of 15 mL min^{-1} for GO suspension.

Linear scanning voltammetry curves of the anodes were detected for the influent with or without GO nanosheets under scanning speed of 2 mV s^{-1}. Electrochemical impedance spectra of the anodes were obtained via AC impedance technique with the potential amplitude of 50 mV over a frequency range of 0.05–50k Hz under the anode potential at 6.0 V.

An atomic force microscope (AFM, Multimode nanoscope, Bruker, USA) was employed to detect the nanosheet configuration and structure. NanoScope Analysis software was used to analyse images and height profiles. For sample preparation, the GO or PGO suspension was dropped on the mica plate and dried at room temperature.

A transmission electron microscope (TEM, JEM-2100, JEOL Ltd.) with accelerating voltage of 200 kV was used to capture the TEM images of nanosheets. For sample preparation, the GO or PGO suspension was dropped on copper mesh-supported carbon film and dried at room temperature.

The morphology of the prepared membranes was observed by using a field-emission scanning electron microscope (SEM, Ultra-55, Zeiss Co.) with accelerating voltage of 5 kV. For fabricating membrane sample and minimizing recharging effect, the membrane was fractured in liquid nitrogen and coated with an ultrathin platinum layer.

X-ray photoelectron spectroscopy (XPS) experiment was carried out by using a RBD (RBD Enterprises, USA) upgraded PHI-5000C ESCA system (Perkin Elmer) with an incident radiation of

monochromatic Mg Kα x-rays ($h\upsilon$ = 1253.6 eV) at 250 W. To ensure sufficient sensitivity and resolution, the high voltage was kept at 14.0 kV, the pass energy was set as 46.95 eV, and the pressure of analysis chamber was below 5×10^{-8} Pa.

For measuring the interlayer space of the membranes, the XRD pattern was collected by using an x-ray diffractometer (XRD, D8 Advance, Bruker Co.) with Cu Kα radiation (λ= 0.154056 nm) in a continuous scanning mode. For dry sample, the membrane was dried at 50 ^{o}C before measurement. For wetted sample, the membrane was immersed in water for 1 h before measurement. The interlayer space was calculated by Bragg's Law of $2d\sin\theta=n\lambda$. Where, d and θ were interlayer spacing and characteristic peak theta angle, respectively; and λ and n were 1.54056 nm and 1, respectively.

Raman spectrum was collected by using a WITEC micro-Raman at 532 nm laser.

A streaming potential analyzer (SurPASS, Anton Paar, Austria) was used to study the zeta potential of the prepared membranes. Potassium chloride solution with concentration of 1 mmol L^{-1} was used as background electrolyte. Hydrochloric acid and sodium hydroxide were used to adjust pH.

Dynamic contact angle of the prepared membranes was recorded by using an optical contact angle and interface tension meter (ST200KB, USA KINO Industry Co.).

Supplementary Discussion

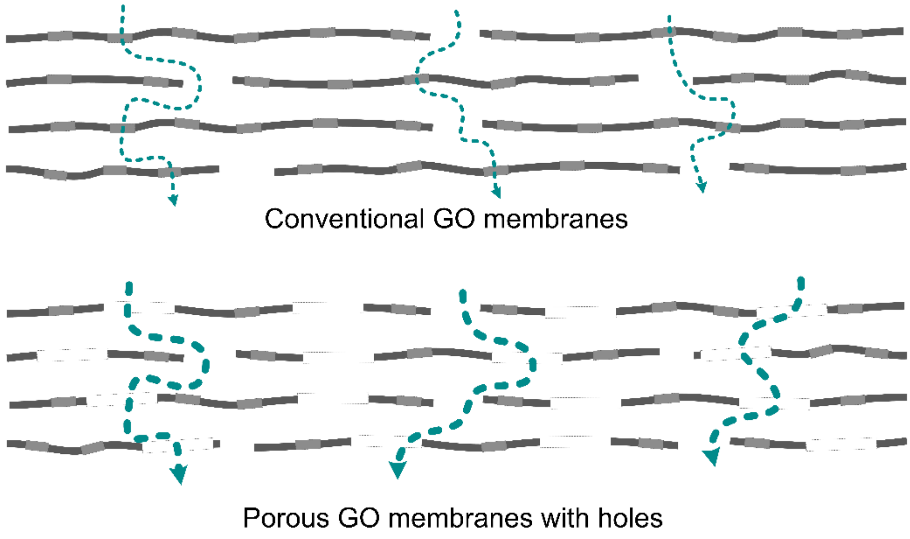

Supplementary Fig. 1. Schematics of transport pathway of the conventional GO membranes and porous GO membranes with holes[1]. Black lines, grey dashed boxes, white dashed boxes, and dark cyan arrows represent GO nanosheets, pinhole defects, perforated holes, and water transport pathways of the GO and PGO membranes. Some wrinkles and voids from a certain irregular stacking may affect molecular transport of GO membranes.

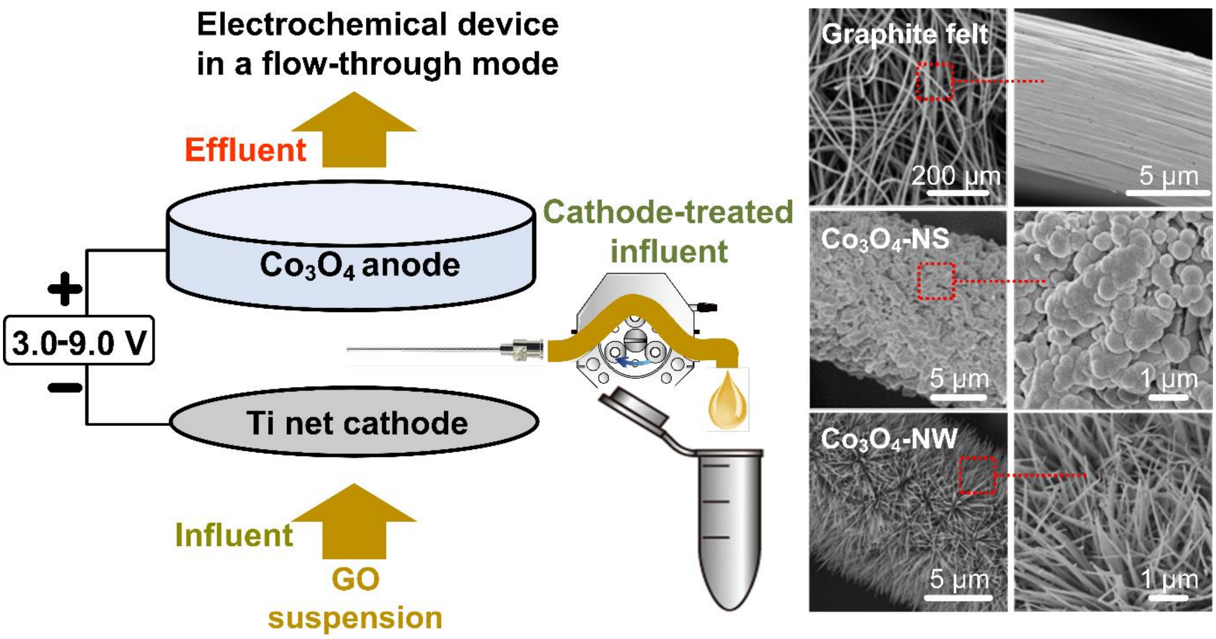

Supplementary Fig. 2. Vertically expanded depiction of electrochemical apparatus in a flow-through mode with in-situ sampling the cathode-treated influent and the SEM images of Co_3O_4-NW and Co_3O_4-NS for perforation. NW and NS represent nanowires and nanospheres, respectively.

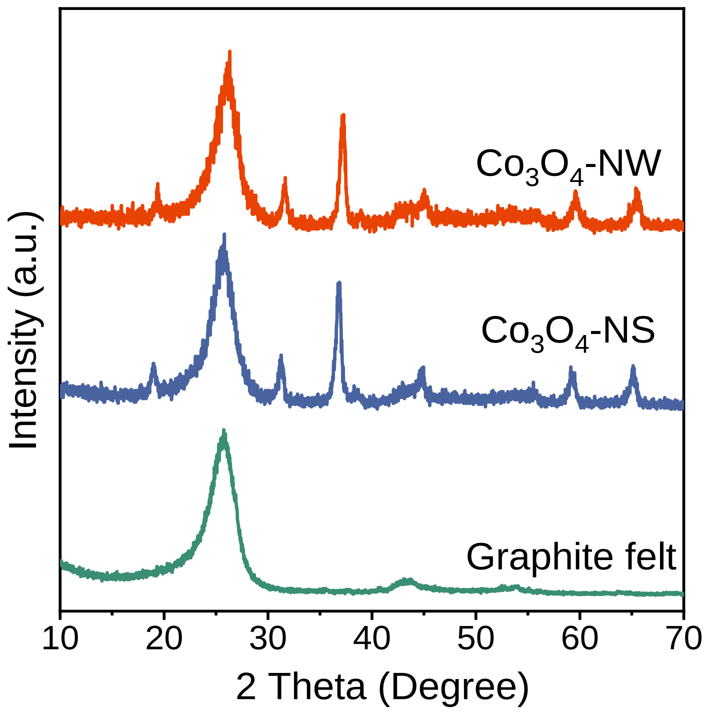

Supplementary Fig. 3. XRD patterns of graphite felt, Co_3O_4-NW and Co_3O_4-NS. The XRD patterns confirmed the crystalline structure of Co_3O_4 with diffraction peaks of 2θ values at 19.1°, 31.4°, 36.7°, 38.5 °, 44.6 °, 55.69 °, 59.3 °, and 65.2 °. a. u. represents arbitrary unit. NW and NS represent nanowires and nanospheres, respectively.

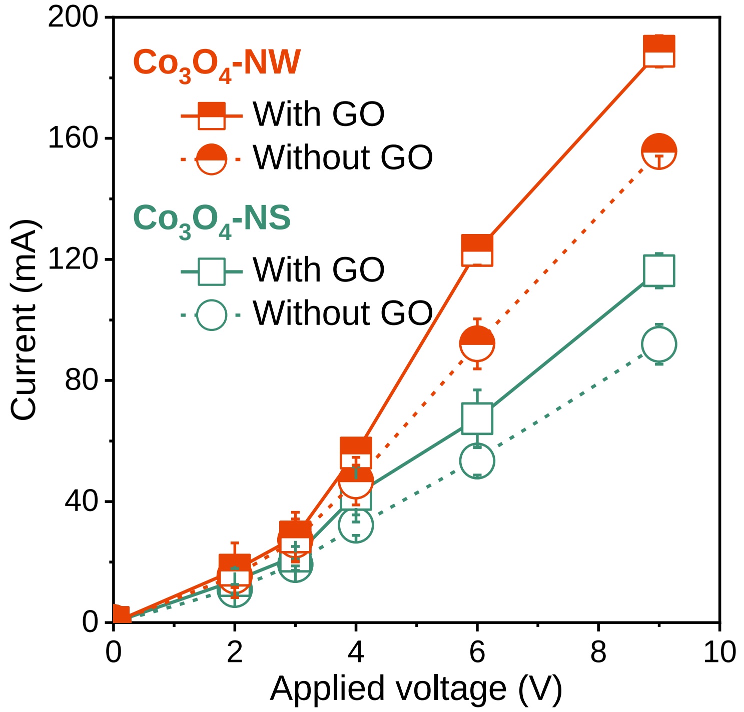

Supplementary Fig. 4. Current of the electrochemical apparatus for treating the solutions with or without GO nanosheets. NW and NS represent nanowires and nanospheres, respectively.

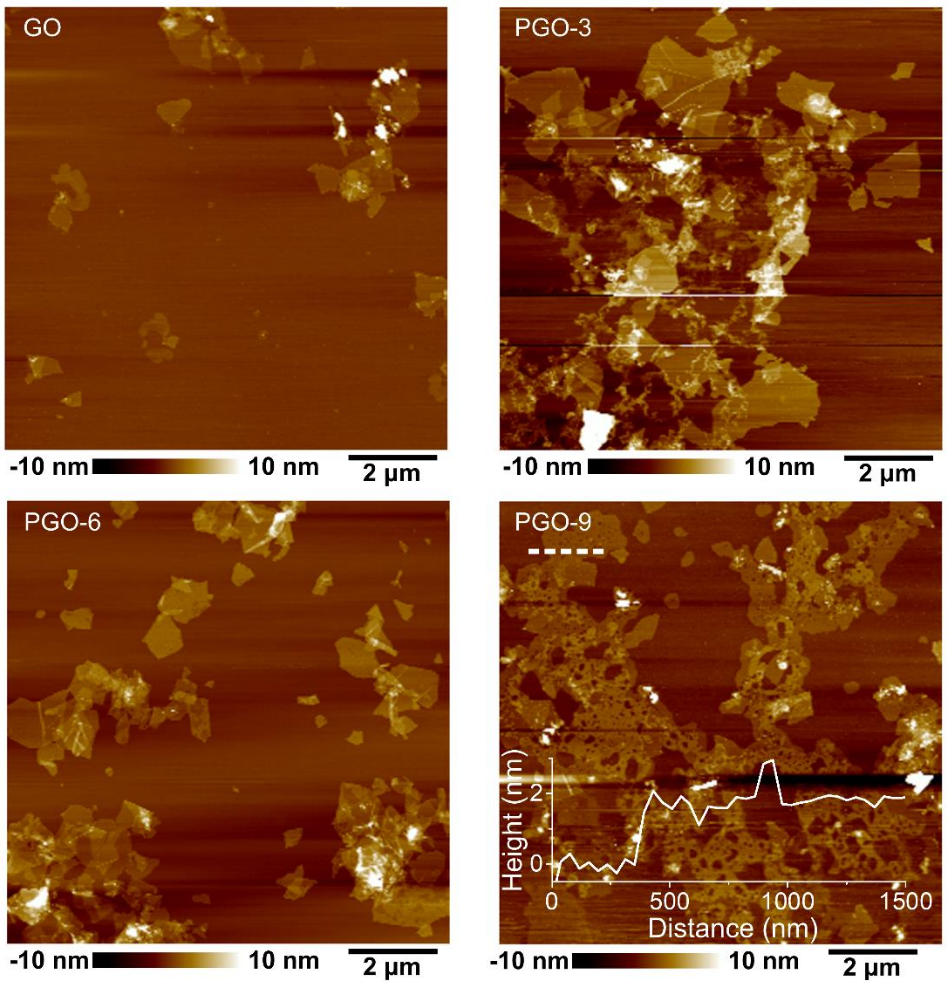

Supplementary Fig. 5. AFM images of the GO, PGO-3, PGO-6, and PGO-9 nanosheets. The height profile of the PGO-9 nanosheets is presented.

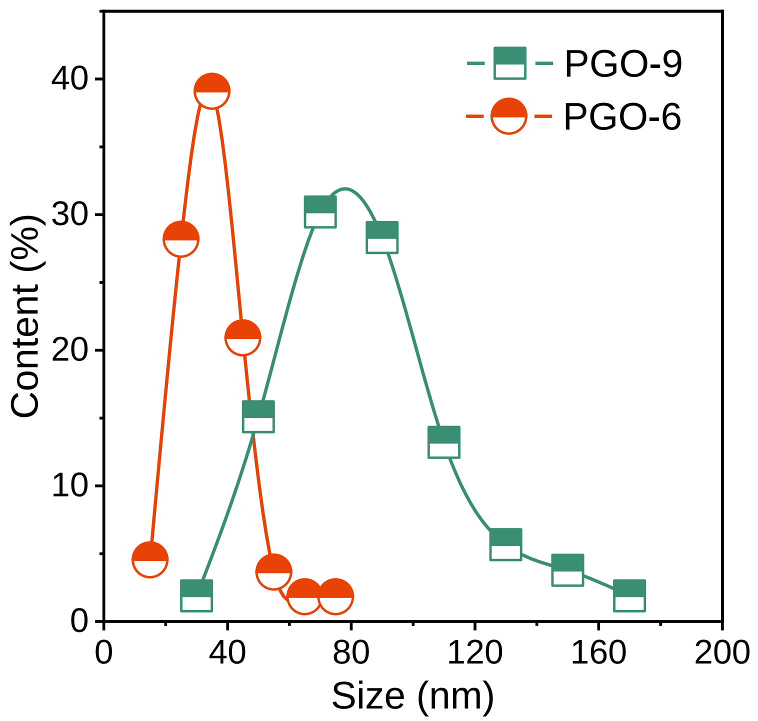

Supplementary Fig. 6. Size distributions of holes in the PGO nanosheets based on the AFM images.

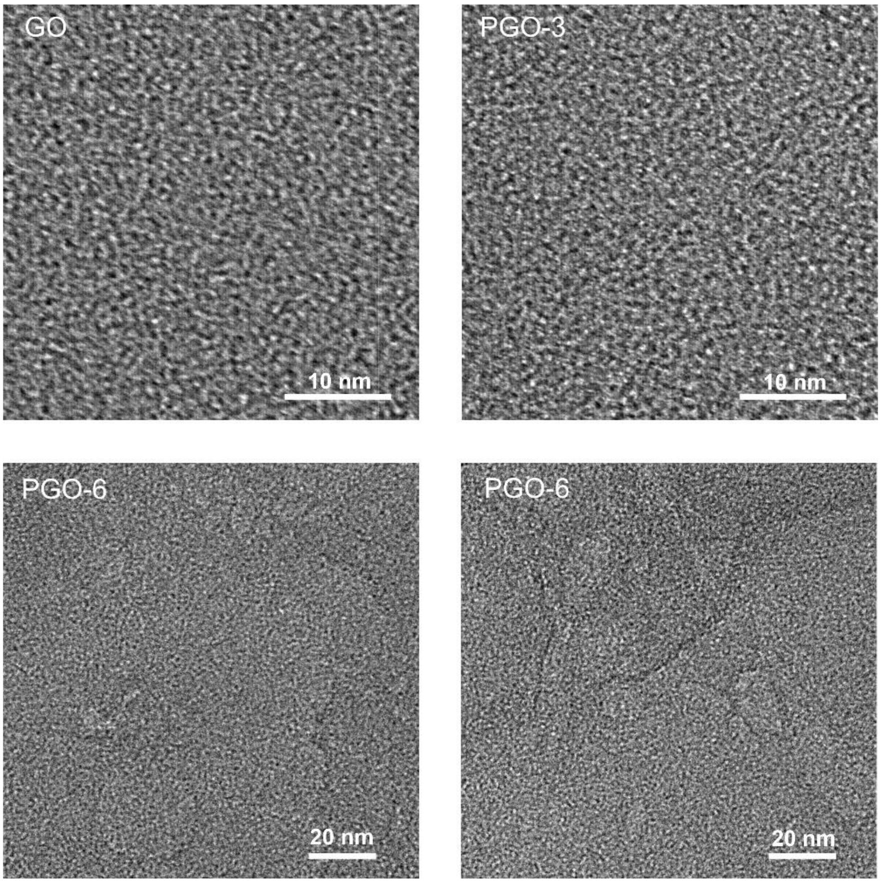

Supplementary Fig. 7. TEM images of the GO, PGO-3, and PGO-6 nanosheets. Some light grey areas with higher transmittance indicated the pores in PGO-6.

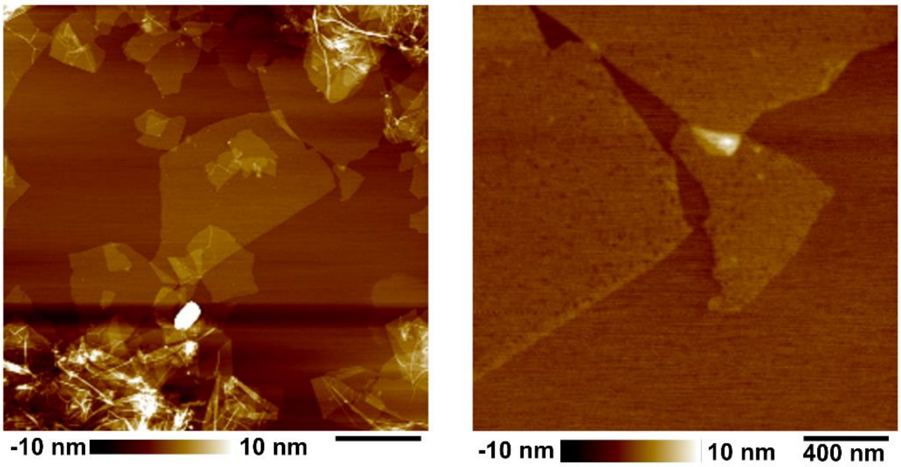

Supplementary Fig. 8. **AFM images of the PGO-6 nanosheets perforated with flow rate of 30 mL min^{-1} and retention time of 4.9 s.**

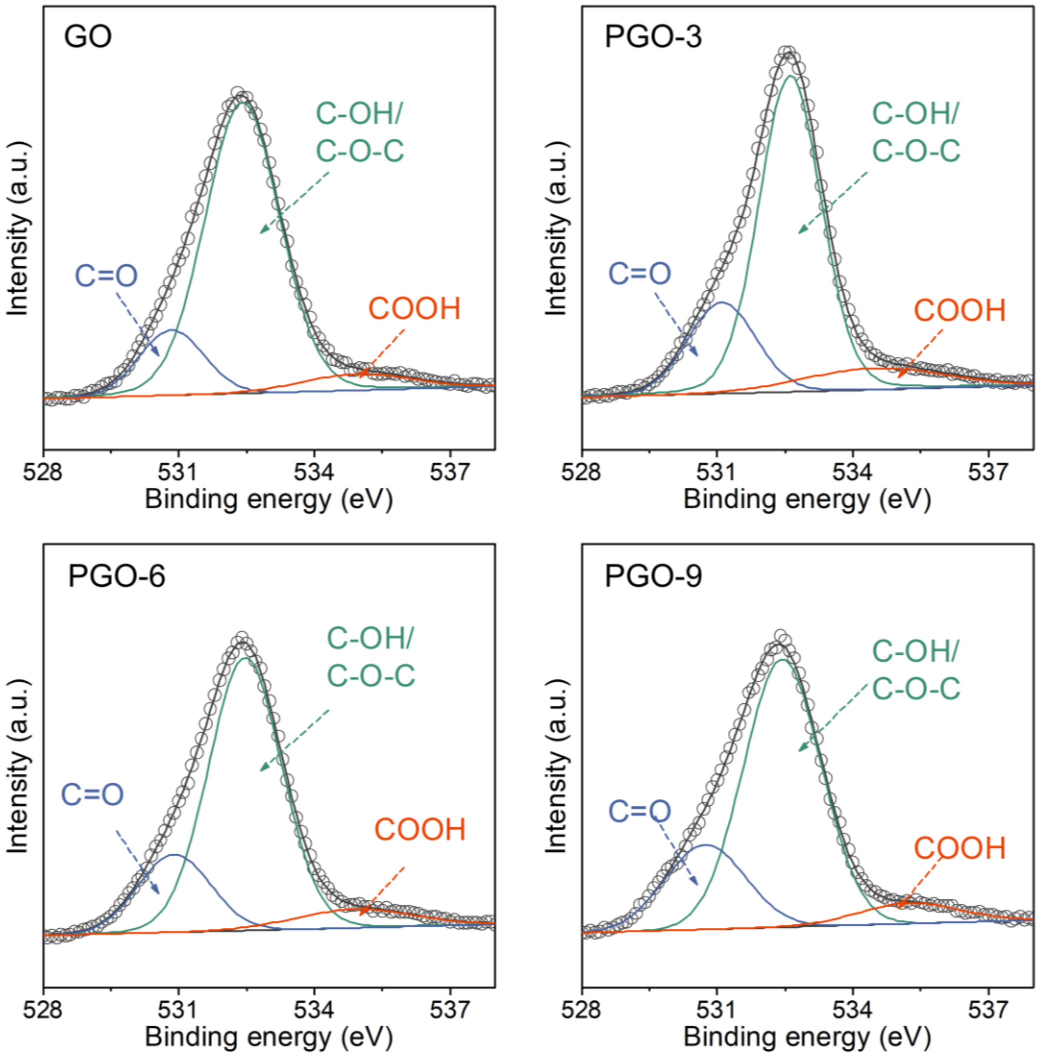

Supplementary Fig. 9. O *1s* XPS spectra of the GO, PGO-3, PGO-6, and PGO-9 nanosheets. a. u. represents arbitrary unit.

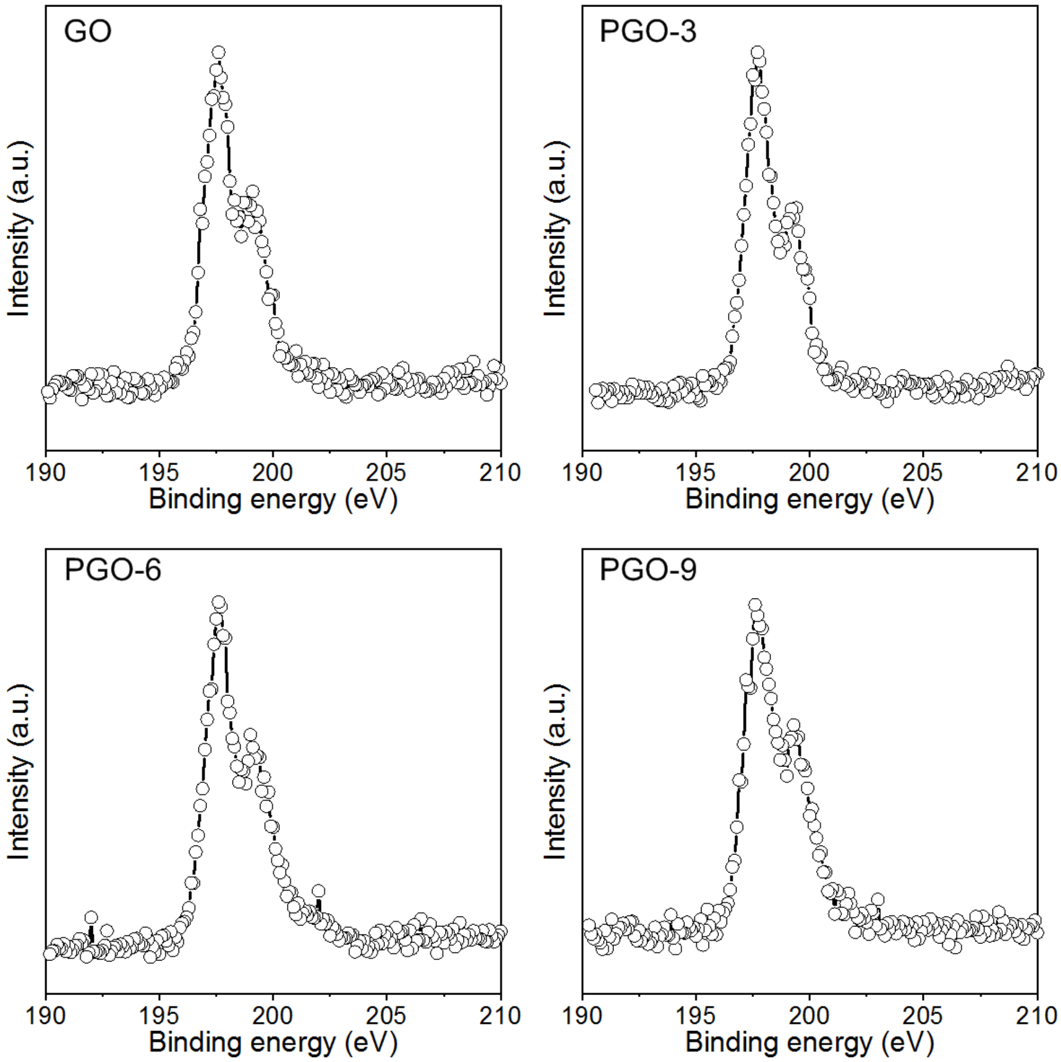

Supplementary Fig. 10. Cl *2p* XPS spectra of the GO, PGO-3, PGO-6, and PGO-9 nanosheets. a. u. represents arbitrary unit. It should be noted that the PGO membranes showed similar chlorine content as GO, suggesting no formation of PGCl. Moreover, no C-Cl in the C *1s* XPS and the similar Cl XPS peak shape for all membranes also indicated that the PGO was not PGOCl.

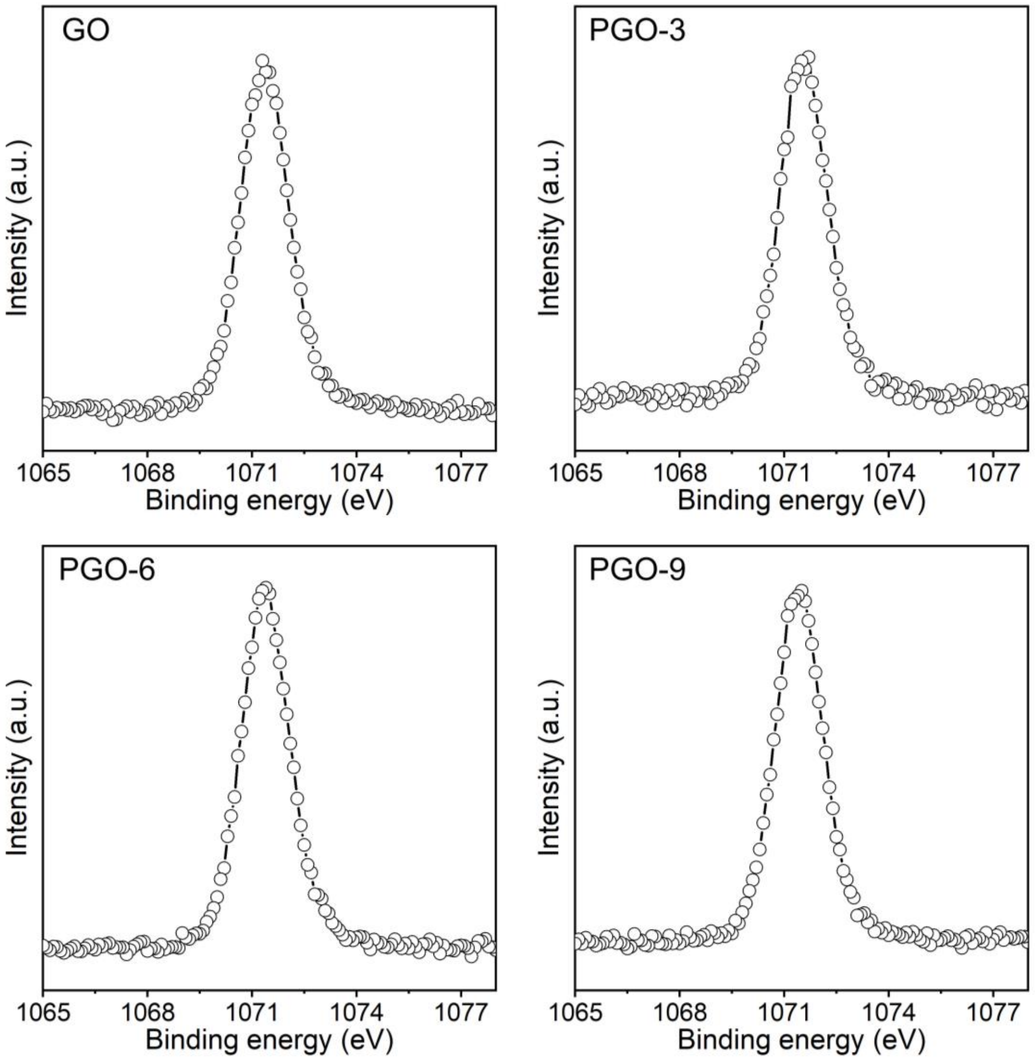

Supplementary Fig. 11. Na *1s* XPS spectra of the GO, PGO-3, PGO-6, and PGO-9 nanosheets. a. u. represents arbitrary unit. The GO, PGO-3, PGO-6, and PGO-9 had sodium and chlorine contents of 3.0–3.5% and 1.5–1.9%, respectively. The more sodium content than chlorine one for all membranes was attributed to the sodium insertion from the interaction between sodium ion and oxygen-containing groups. The sodium ion insertion would be beneficial to the stability of the GO and PGO membranes in nanofiltration[2].

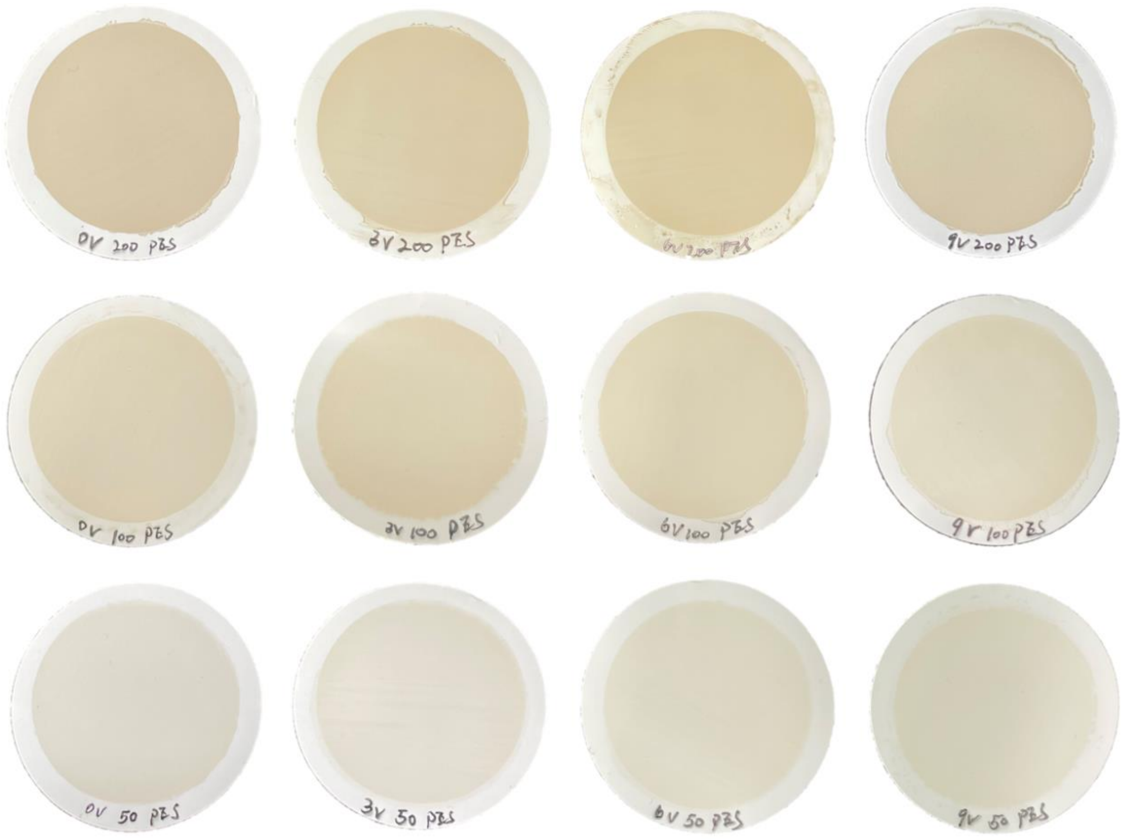

Supplementary Fig. 12. Photographs of the GO, PGO-3, PGO-6, and PGO-9 membranes with different loadings of 200, 100, and 50 μg. The membranes were prepared with diameter of 4.2 cm on polyethersulfone (PES) substrates. Photo Credit: W.L., Jinan University.

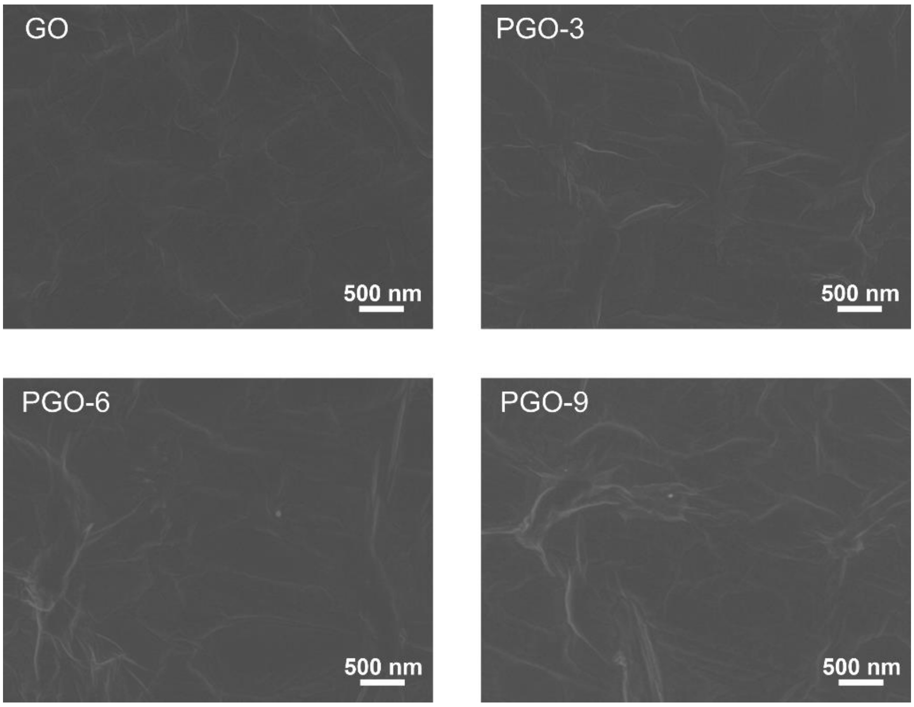

Supplementary Fig. 13. Top view SEM images of the GO, PGO-3, PGO-6, and PGO-9 membranes prepared with loading of 200 μg.

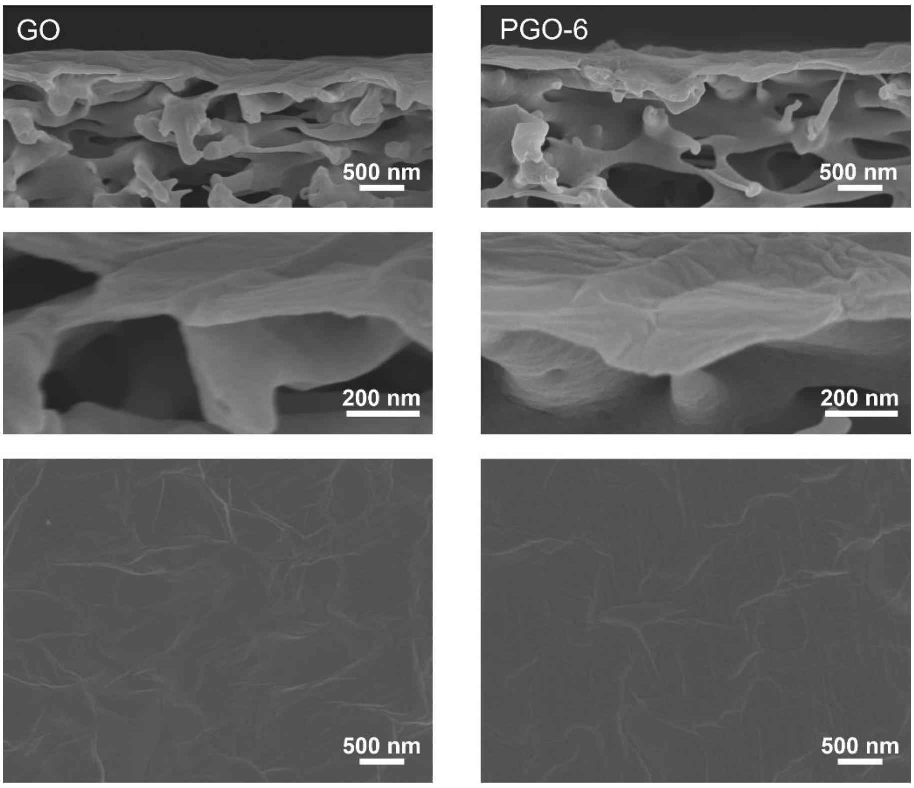

Supplementary Fig. 14. Cross-sectional and top view SEM images of the GO and PGO-6 membranes prepared with loading of 100 µg.

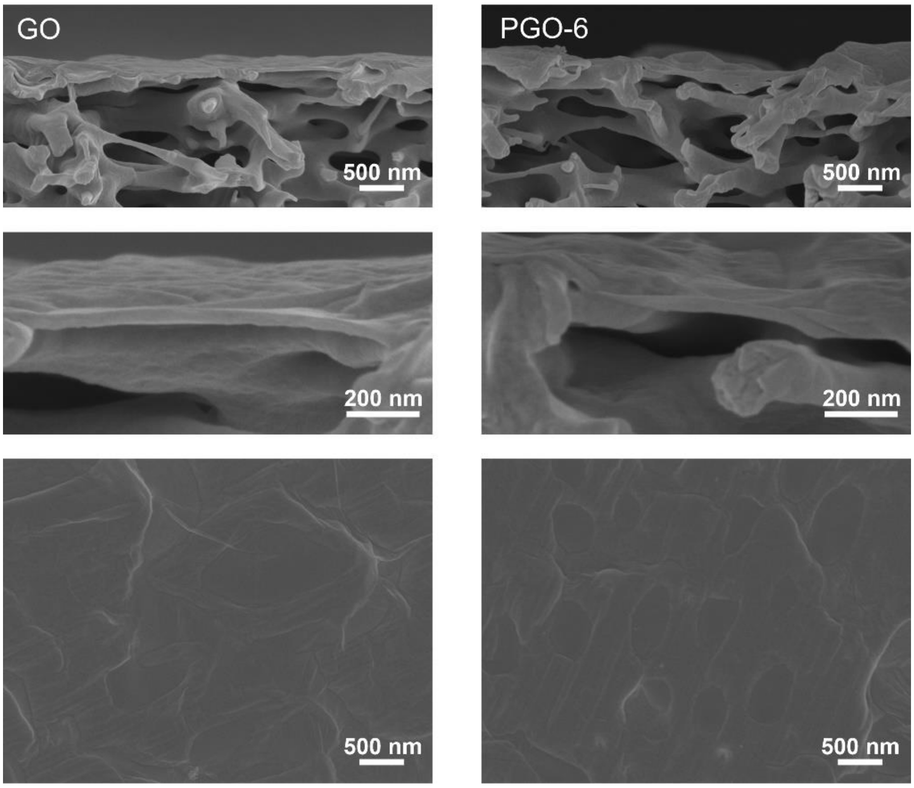

Supplementary Fig. 15. Cross-sectional and top view SEM images of the GO and PGO-6 membranes prepared with loading of 50 μg.

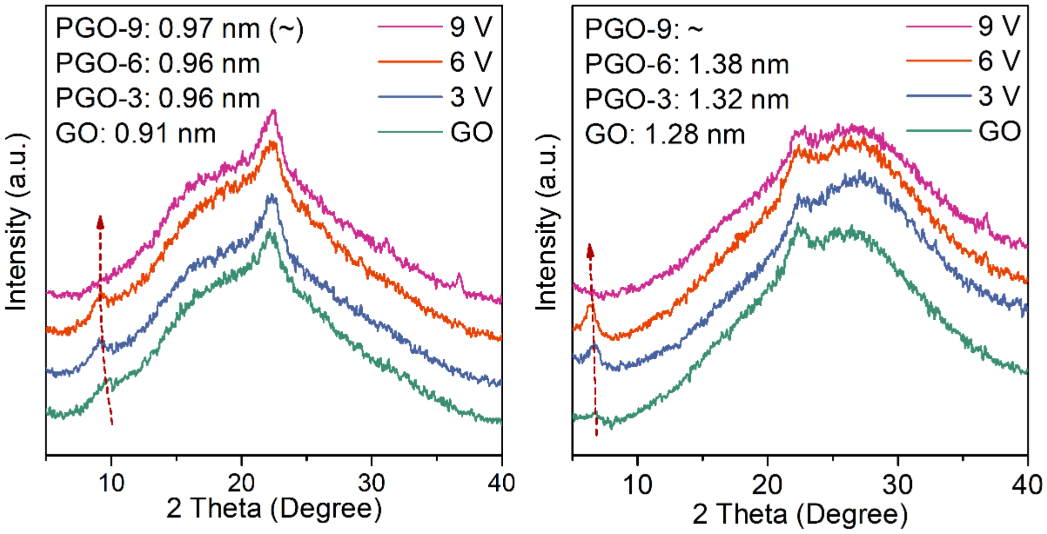

Supplementary Fig. 16. **XRD patterns of the GO, PGO-3, PGO-6, and PGO-9 membranes at dry and wetted states.** a. u. represents arbitrary unit.

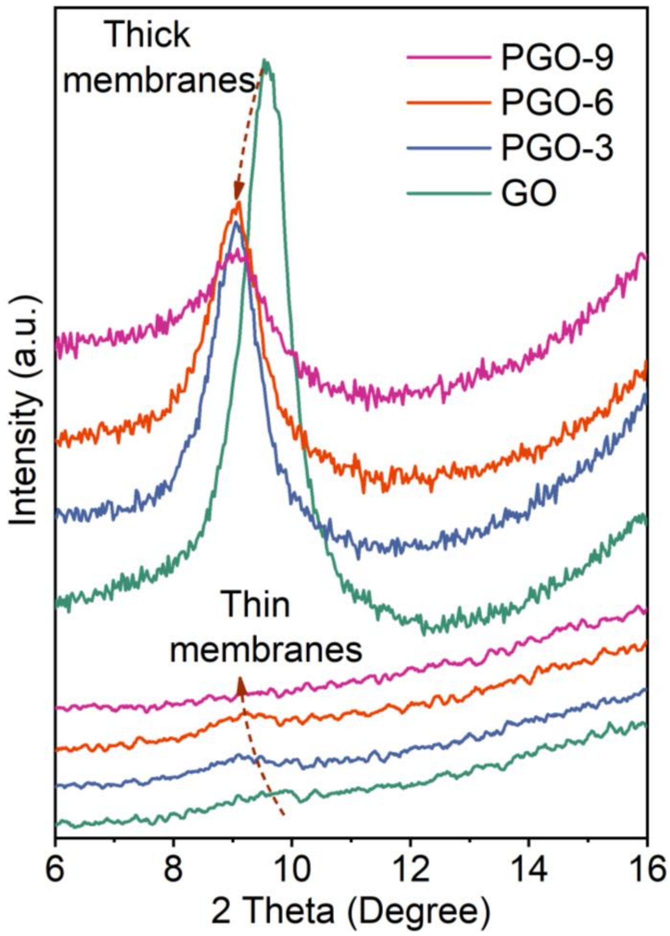

Supplementary Fig. 17. **XRD patterns of the GO, PGO-3, PGO-6, and PGO-9 membranes with loading of 1600 μg.** a. u. represents arbitrary unit. The XRD patterns (also shown in Fig. 4e) of the thin GO and PGO membranes with loading of 200 μg are presented for comparison. The XRD peak position of the thick membranes was similar to those of the thin membranes. After electrochemical perforation, the XRD peak of PGO shifted to lower degree compared with that of GO, suggesting the expanded interlayer space. Although the thick PGO-9 membrane with characteristic peak was different from the thin PGO-9 membrane with almost no XRD peak, the peak intensity of the thick PGO-9 membrane was much smaller than that of other thick membranes due to the irregular nanosheet arrangement.

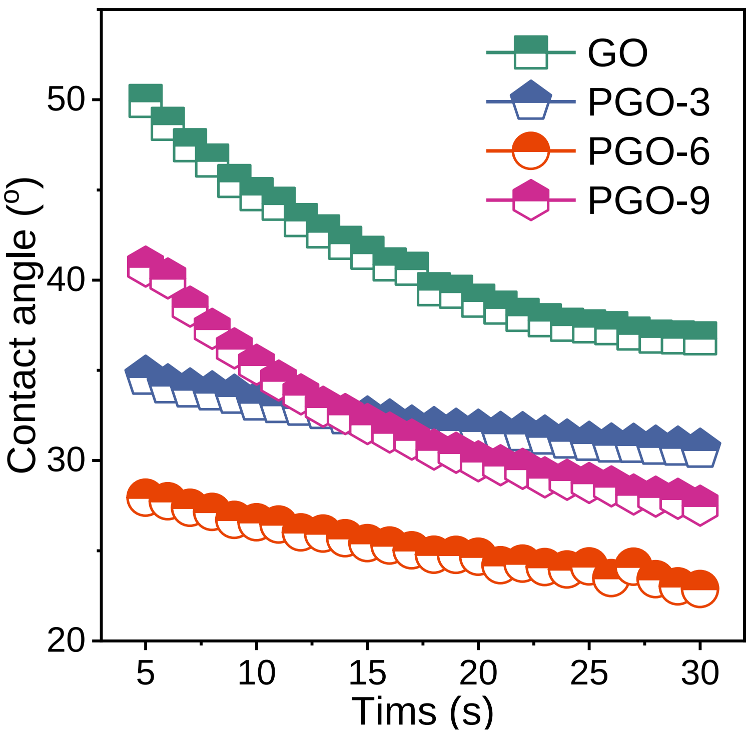

Supplementary Fig. 18. Dynamic water contact angles of the GO, PGO-3, PGO-6, and PGO-9 membranes prepared with loading of 200 μg.

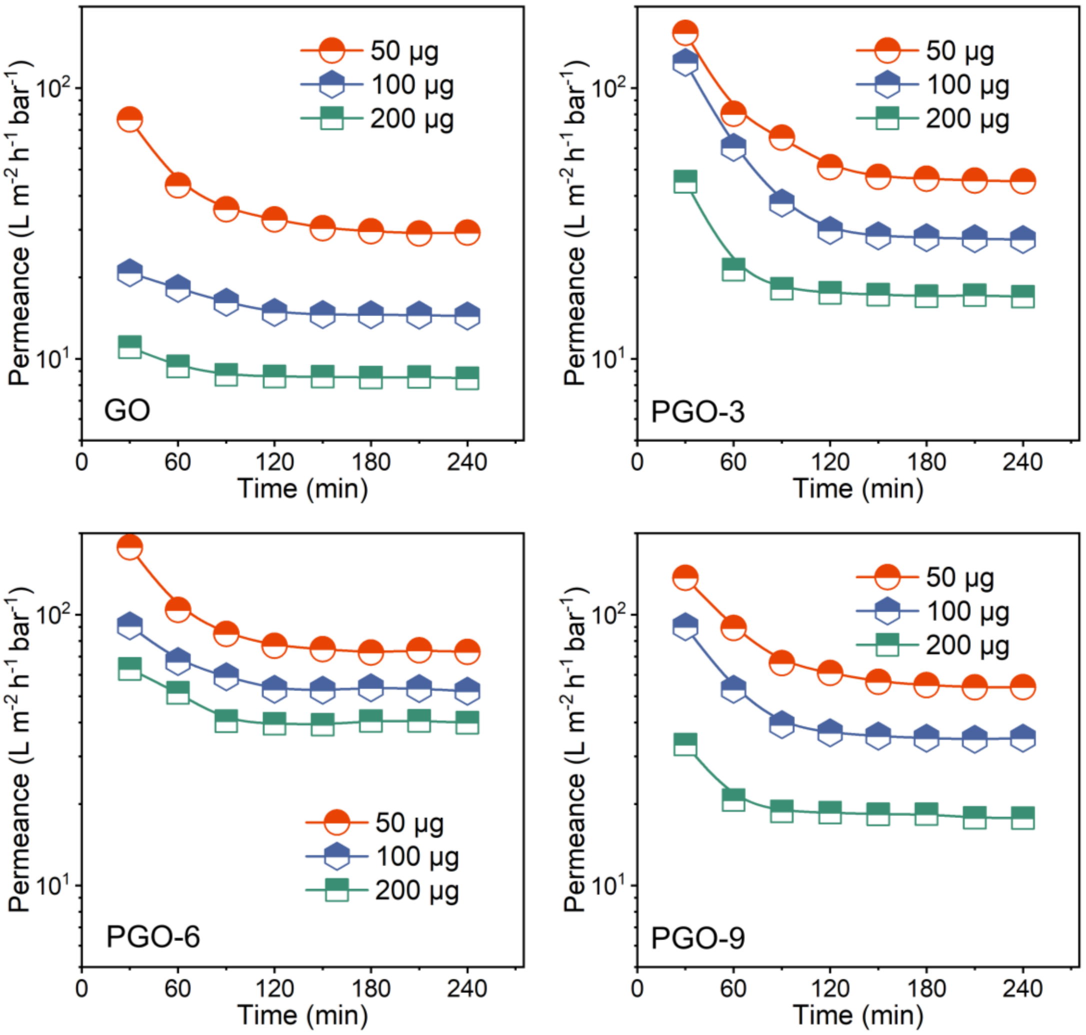

Supplementary Fig. 19. Initial permeance change during filtration of the GO, PGO-3, PGO-6, and PGO-9 membranes with different loadings of 200, 100, and 50 μg. The membrane permeance rapidly decreased at the beginning and then stabilized at room temperature and feed pressure of 2 bar.

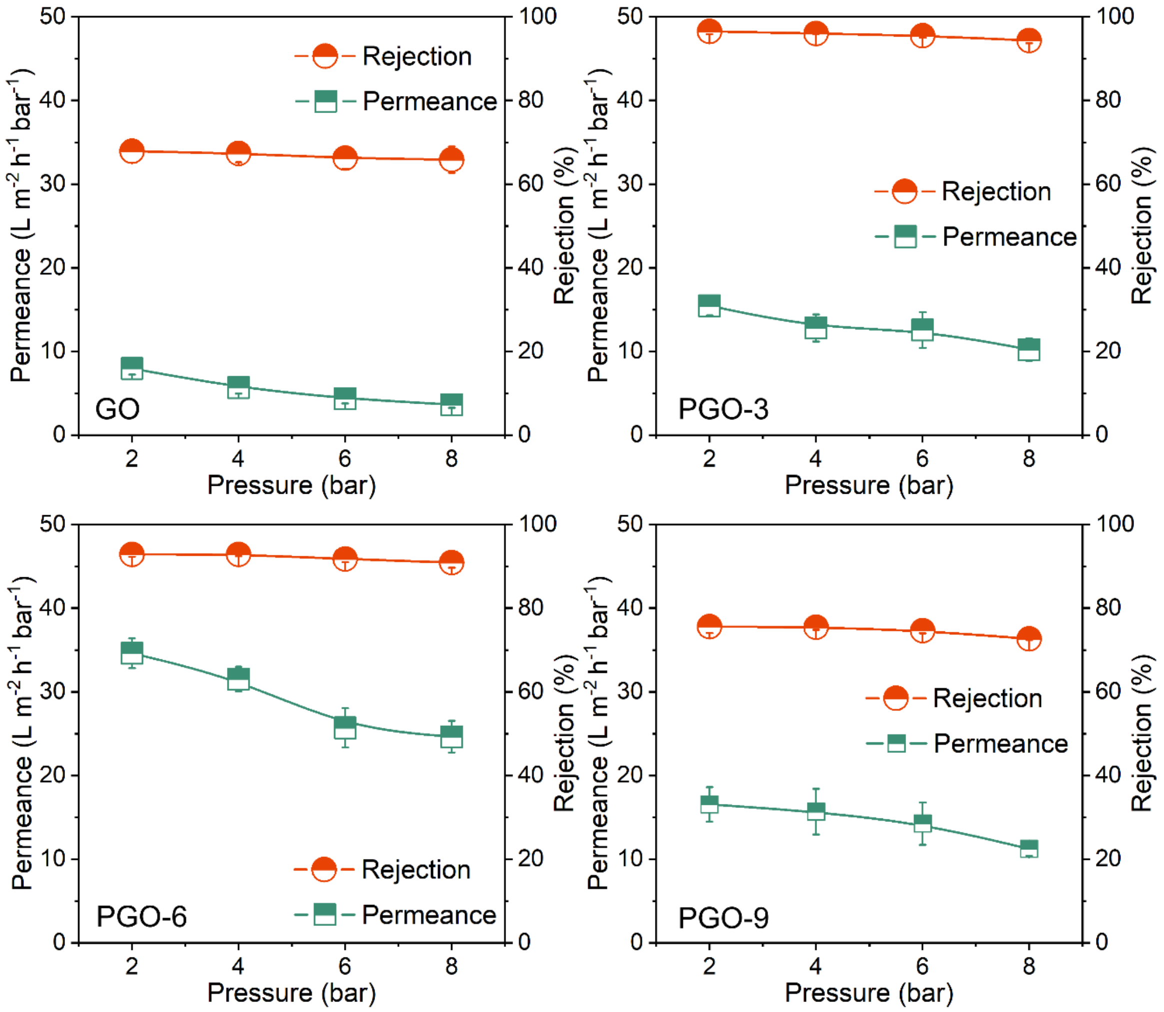

Supplementary Fig. 20. Water permeance and Na_2SO_4 rejection of the GO, PGO-3, PGO-6, and PGO-9 membranes prepared with loading of 200 μg at room temperature and different feed pressures. Error bars are standard deviations from three membrane samples.

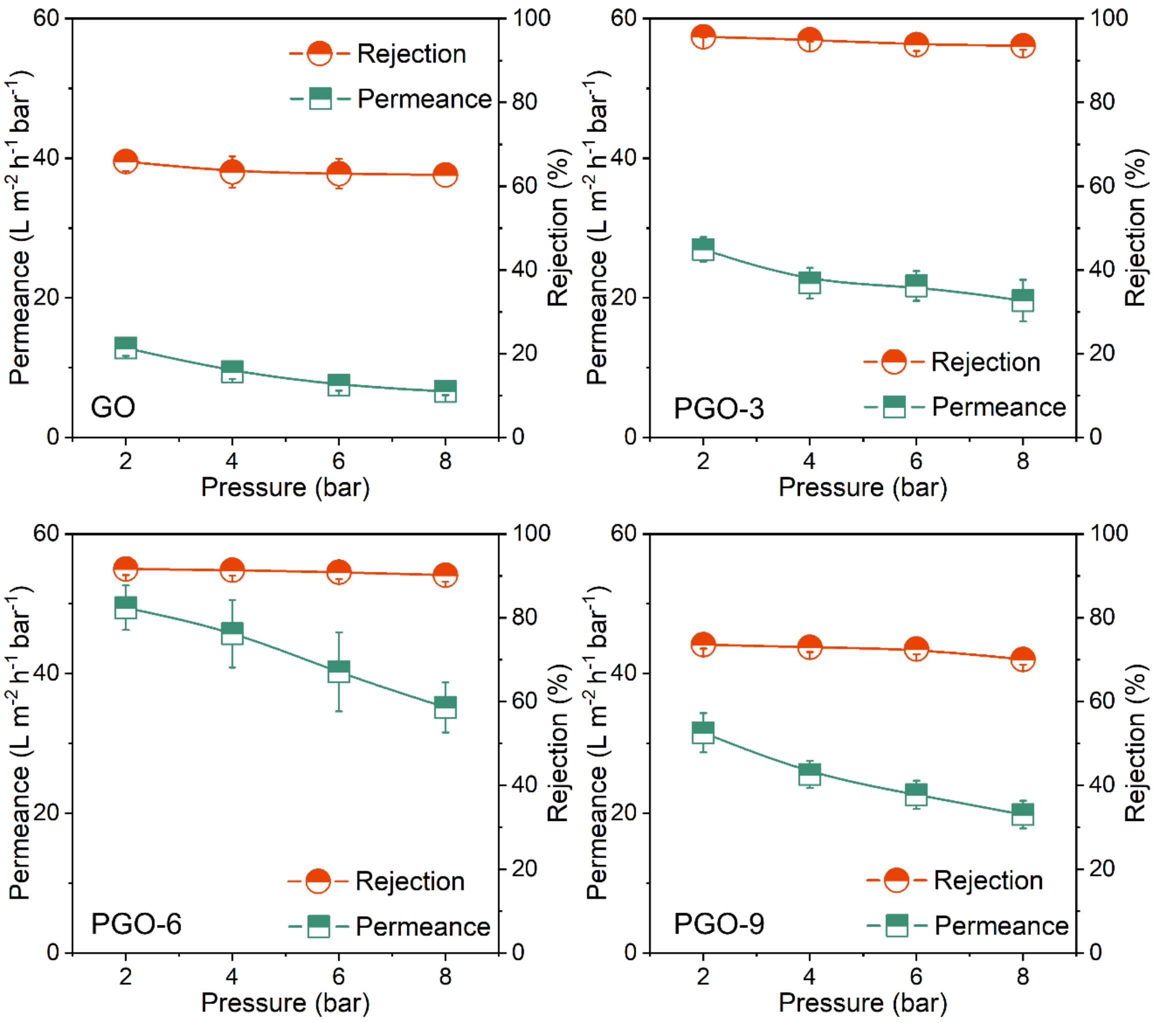

Supplementary Fig. 21. Water permeance and Na_2SO_4 rejection of the GO, PGO-3, PGO-6, and PGO-9 membranes prepared with loading of 100 μg at room temperature and different feed pressures. Error bars are standard deviations from three membrane samples

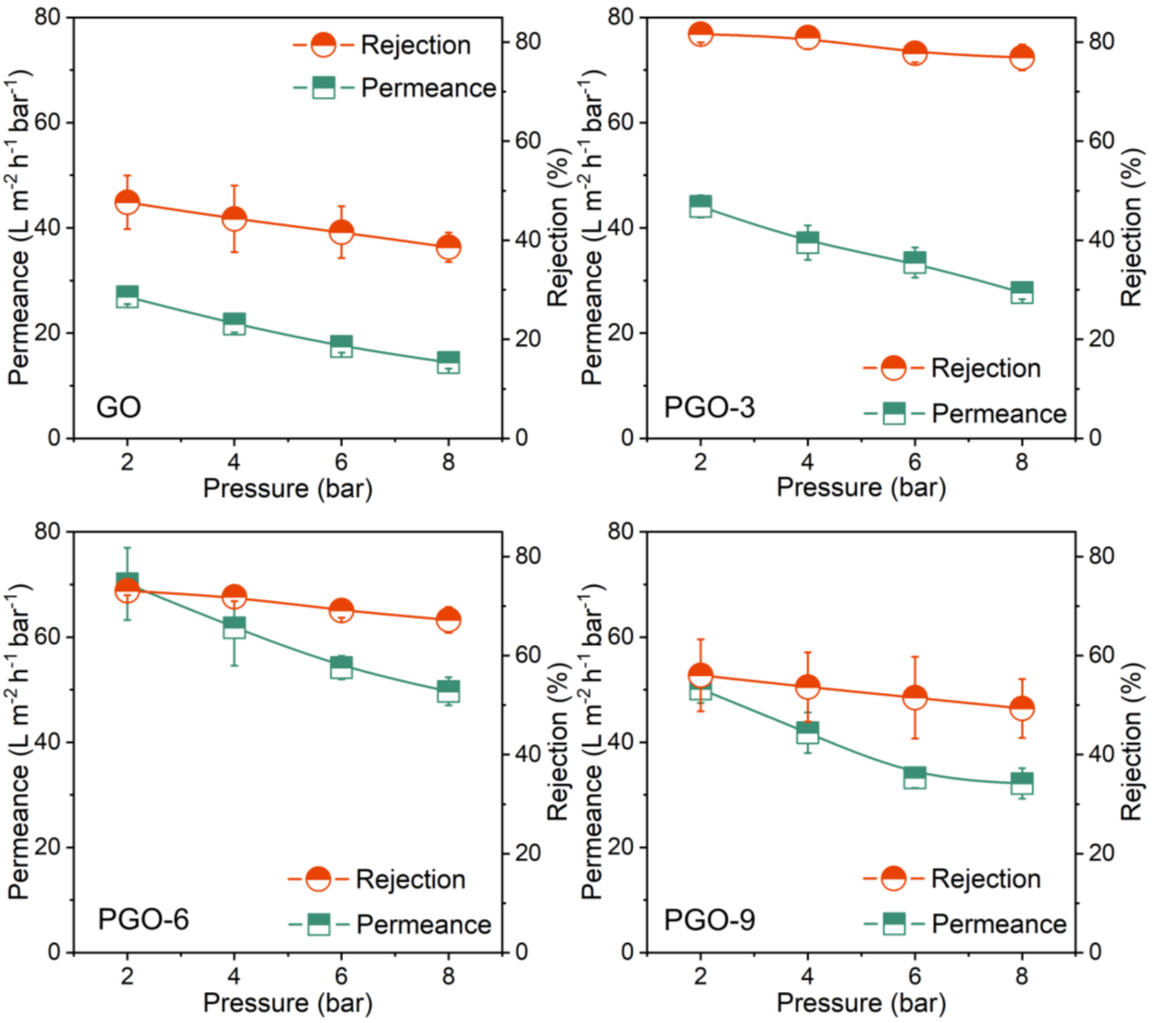

Supplementary Fig. 22. Water permeance and Na_2SO_4 rejection of the GO, PGO-3, PGO-6, and PGO-9 membranes prepared with loading of 50 μg at room temperature and different feed pressures.

Error bars are standard deviations from three membrane samples

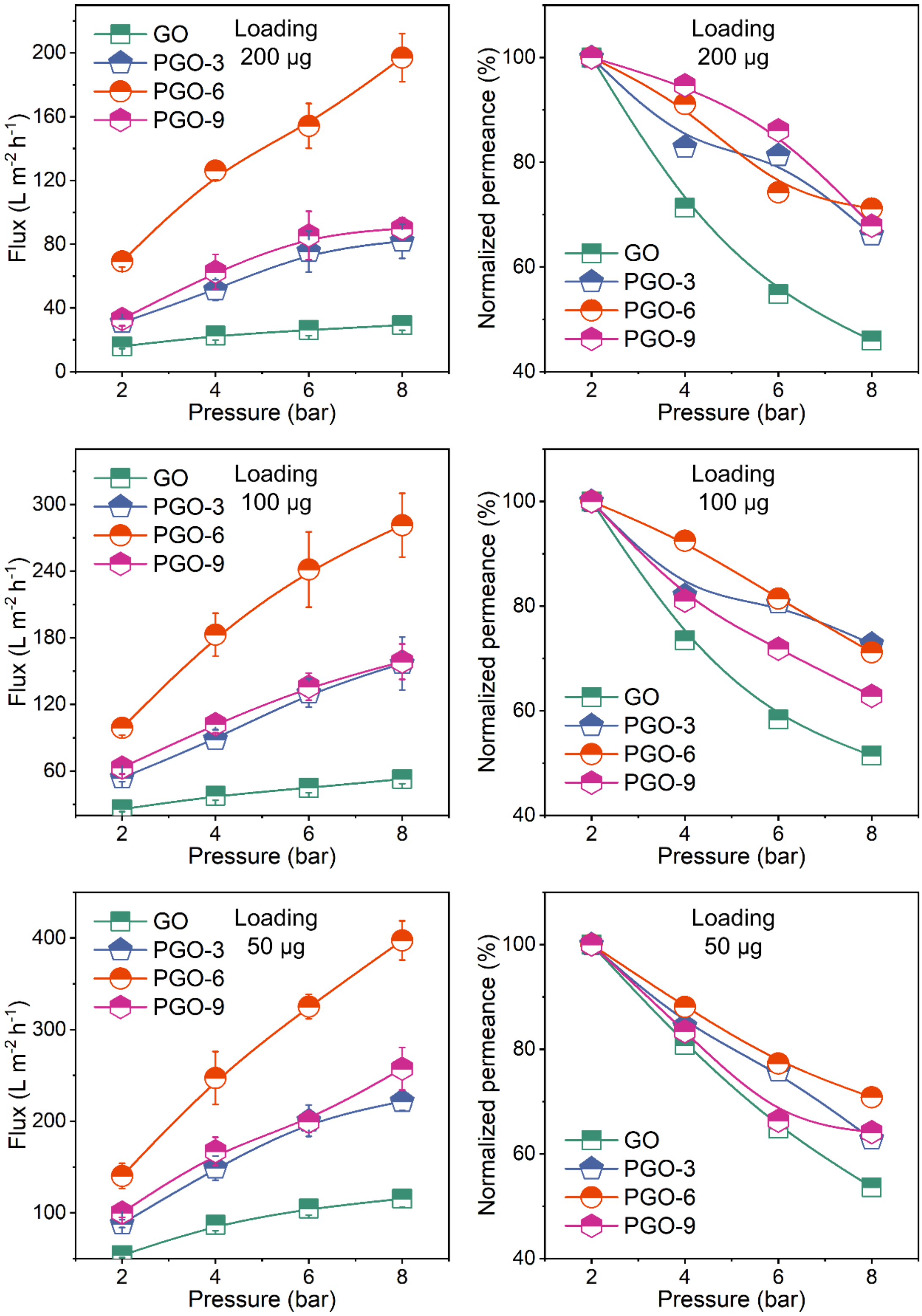

Supplementary Fig. 23. Water flux and normalized permeance of the GO, PGO-3, PGO-6, and PGO-9 membranes prepared with different loadings at different feed pressures. Error bars are standard deviations from three membrane samples.

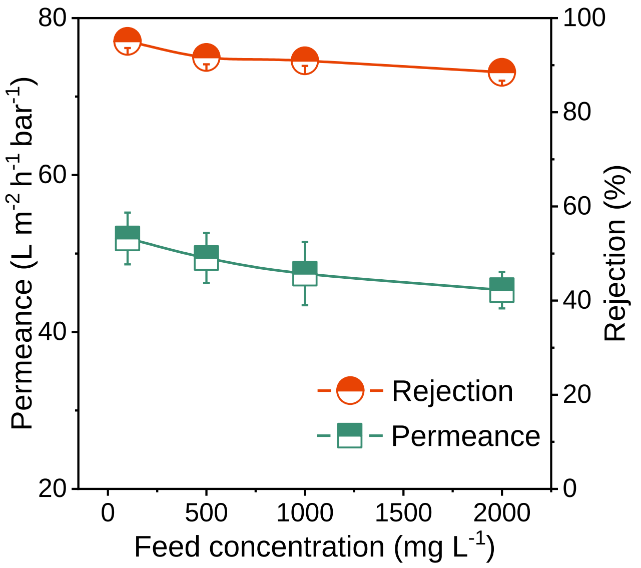

Supplementary Fig. 24. Water permeance and rejection of the PGO membrane prepared with loading of 100 µg for Na_2SO_4 solution with different concentrations. Error bars are standard deviations from three membrane samples.

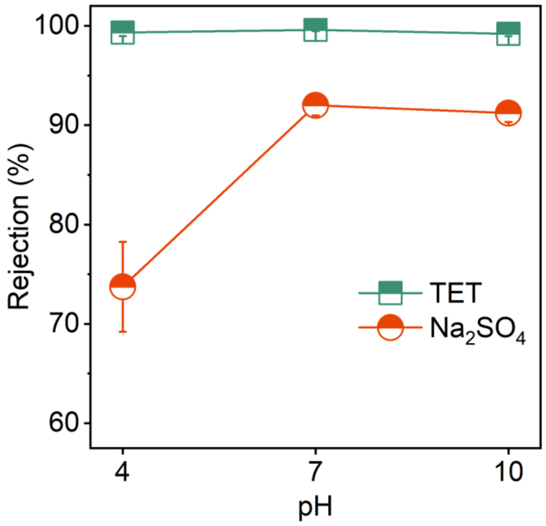

Supplementary Fig. 25. Rejection of the PGO-6 membrane prepared with loading of 100 μg for Na_2SO_4 and TET (tetracycline) solutions with different pH at pressure 2 bar. The Na_2SO_4 rejection decreased at pH of 4 due to the reduction of Donnan effect. However, for tetracycline removal, the separation efficiency was kept at high level under different pH due to the size exclusion separation mechanism. Error bars are standard deviations from three membrane samples.

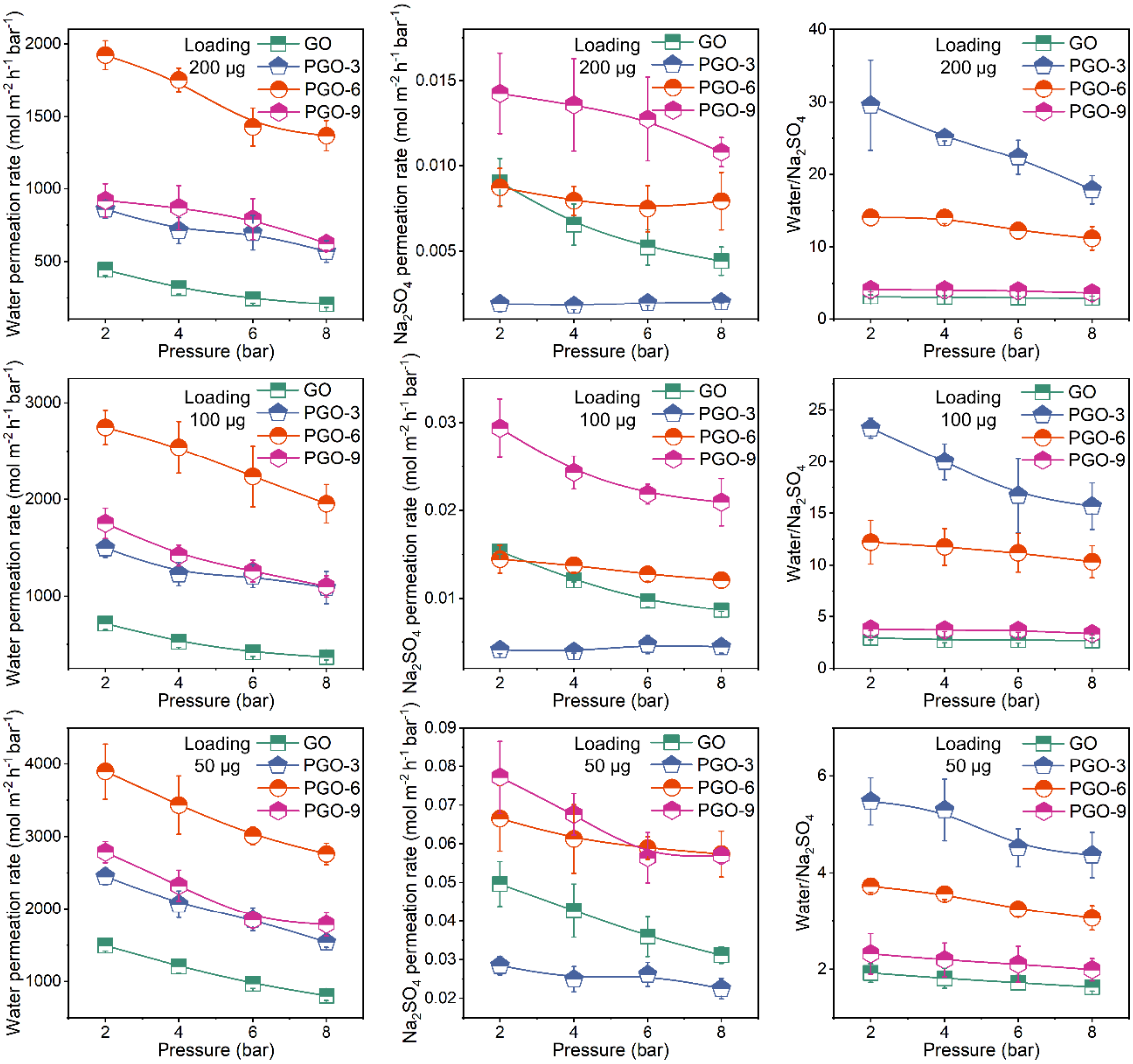

Supplementary Fig. 26. Water permeation rate, Na_2SO_4 permeation rate, and water/Na_2SO_4 selectivity of the GO and PGO membranes prepared with different loadings at different feed pressures. Error bars are standard deviations from three membrane samples.

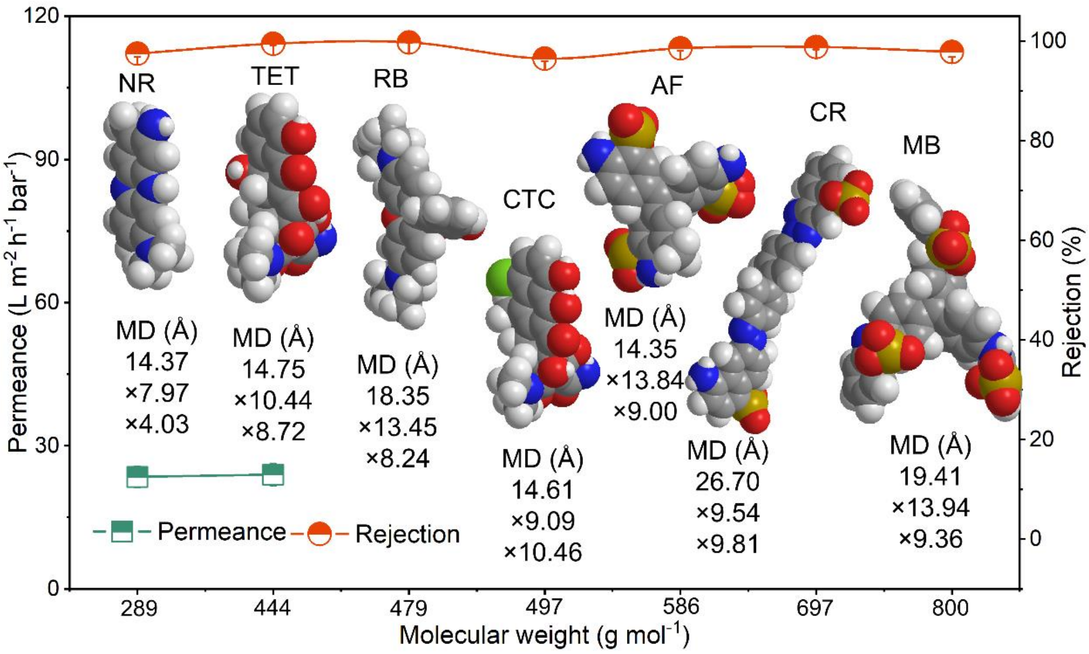

Supplementary Fig. 27. Separation performance of the GO membrane prepared with loading of 50 μg for antibiotic remediation and dye removal at pressure 2 bar. NR, TET, RB, CTC, AF, CR, and MB are neutral red, tetracycline, rhodamine B, chlortetracycline hydrochloride, acid fuchsin, congo red, and methyl blue, respectively. Molecular dimensions (MD) are presented. Error bars are standard deviations from three membrane samples.

Supplementary Table 1. C/O ratio, C-H/C-C/C=C, C-OH/C-O-C, C=O, and COOH contents of the GO and PGO nanosheets from C *1s* and O *1s* XPS spectra.

Nanosheet	C/O ratio	C-H/C-C/C=C	C-OH/C-O-C	C=O	COOH
GO	2.46	60.26	31.44	5.85	2.45
PGO-3	1.96	48.84	35.20	10.82	5.14
PGO-6	2.34	57.63	30.61	8.47	3.29
PGO-9	2.25	57.05	30.10	9.85	3.00

Supplementary Table 2. Comparison between nanowire electrochemical perforation and other methods. RT: Room temperature; and NMP: N-methylpyrrolidone.

Method	Operation	Reagent	Condition	Raw	C/O	Ref.
Chemical etching	Batch	NH_4OH and H_2O_2	50 ℃ for 1–5 h	Liquid	Higher	3
Chemical etching	Batch	H_2O_2	100 ℃ for 1–4 h	Liquid	-	4
Chemical etching	Batch	$KMnO_4$, oxalic acid, and hydrochloric acid	RT for ~6 h	Liquid	Similar	5
Chemical etching	Batch	H_2O_2	70 ℃ for10 h	Liquid	Similar	6
Air oxidation processing graphene and oxidation	Batch	Air	395–460 ℃ for 10 h	Solid	-	7,8
Thermal annealing and microwave treatment	Batch	Air and NMP	200 °C for 10 min	Solid	Higher	9
Nanowire electrochemical perforation	Flow-through	NaCl	RT	Liquid	Lower or similar	This work

Supplementary Table 3. Interlayer space (IS), I_D/I_G ratio, and I_{2D}/I_{D+G} ratio of the GO and PGO membranes.

Membrane	IS (dry, nm)	IS (wet, nm)	I_D/I_G	I_{2D}/I_{D+G}
GO	0.91	1.28	0.83	0.84
PGO-3	0.96	1.32	0.84	0.74
PGO-6	0.96	1.38	0.83	0.70
PGO-9	0.97	-	0.81	0.67

Supplementary Table 4. Separation performance of some recently reported GO membranes after modification with advanced performance. TBO: toluidine blue O; SA: sodium alginate; PAA: polyacrylic acid; PSS: polystyrene sulfonate; rHGO: reduced holey GO membranes; and rNPGO: reduced nanoporous GO membrane.

Membrane	Solute	Concentration (ppm)	Permeance ($L\ m^{-2}\ h^{-1}\ bar^{-1}$)	Rejection (%)	Ref
rGO	Na_2SO_4	2000	6	96	5
ZIF-8@GO	Na_2SO_4	100	49.8	52.9	10
NF-270	Na_2SO_4	100	10.1	91.5	10
GO	Na_2SO_4	100	2.1	84.3	10
GO	Na_2SO_4	14200	0.7	84	11
GO+9.1wt% TBO	Na_2SO_4	14200	0.4	90	11
GO-SA	Na_2SO_4	50	20.2	88.4	12
GO-PAA	Na_2SO_4	50	14.3	95.3	12
GO-PSS	Na_2SO_4	50	16.8	97.1	12
rHGO	Na_2SO_4	2000	9.2	96.7	4
rHGO	Na_2SO_4	2000	14.0	91.1	4
rNPGO	Na_2SO_4	2840	39.9	90.0	6

Supplementary References

1. Saraswat, V. et al. Invariance of water permeance through size-differentiated graphene oxide laminates. *ACS Nano* **12**, 7855-7865 (2018).
2. Chen, L. et al. Ion sieving in graphene oxide membranes via cationic control of interlayer spacing. *Nature* **550**, 380-383 (2017).
3. Wu, T., Moghadam, F. & Li, K. High-performance porous graphene oxide hollow fiber membranes with tailored pore sizes for water purification. *J. Membr. Sci.* **645**, 120216 (2022).
4. Chen, X. et al. Reduced holey graphene oxide membranes for desalination with improved water permeance. *ACS Appl. Mater. Interfaces* **12**, 1387-1394 (2020).
5. Ying, Y., Sun, L., Wang, Q., Fan, Z. & Peng, X. In-plane mesoporous graphene oxide nanosheet assembled membranes for molecular separation. *RSC Adv.* **4**, 21425-21428 (2014).
6. Li, Y. et al. Thermally reduced nanoporous graphene oxide membrane for desalination. *Environ. Sci. Technol.* **53**, 8314-8323 (2019).
7. Buelke, C. et al. Evaluating graphene oxide and holey graphene oxide membrane performance for water purification. *J. Membr. Sci.* **588**, 117195 (2019).
8. Lin, Y. et al. Holey graphene nanomanufacturing: structure, composition, and electrochemical properties. *Adv. Funct. Mater.* **25**, 2920-2927 (2015).
9. Kang, J. et al. Microwave-assisted design of nanoporous graphene membrane for ultrafast and switchable organic solvent nanofiltration. *Nat. Commun.* **14**, 901 (2023).
10. Zhang, W. H. et al. Graphene oxide membranes with stable porous structure for ultrafast water transport. *Nat. Nanotechnol.* **16**, 337-343 (2021).
11. Wang, Z. et al. Graphene oxide nanofiltration membranes for desalination under realistic conditions.

Nat. Sustain. **4**, 402-408 (2021).

12. Zhang, M. et al. Controllable ion transport by surface-charged graphene oxide membrane. *Nat. Commun.* **10**, 1253 (2019).
